# Endovascular Treatment of Unruptured Wide Necked Cerebral Aneurysms Larger Than 9 mm Affects Re-treatment and Prognosis in the Elderly: A Retrospective Analysis of Unruptured Aneurysms

**DOI:** 10.7759/cureus.75759

**Published:** 2024-12-15

**Authors:** Shuto Fushimi, Taisuke Akimoto, Yuta Otomo, Yu Iida, Shigeta Miyake, Makoto Ohtake, Satoshi Hori, Jun Suenaga, Yasunobu Nakai, Tetsuya Yamamoto

**Affiliations:** 1 Neurosurgery, Yokohama City University Graduate School of Medicine, Yokohama, JPN; 2 Neurosurgery, Yokohama City University Medical Center, Yokohama, JPN; 3 Neurosurgery, Yokohama Brain and Spine Center, Yokohama, JPN

**Keywords:** elderly, endovascular treatment, prognostic risk factors, unruptured cerebral aneurysm, wide cerebral aneurysms

## Abstract

Background and purpose

The risk of rupture increases with advancing age. However, the risk-benefit of coil embolization for elderly patients with unruptured aneurysms is controversial. This study aimed to identify factors associated with treatment primary outcomes, including risk factors for complications and aneurysm recurrence requiring re-treatment in the endovascular treatment of unruptured aneurysms in elderly patients. In addition, deterioration of the modified Rankin Scale (mRS) was examined as a secondary outcome.

Materials and methods

This retrospective three-center study examined 112 cases of coiled unruptured aneurysms in patients aged ≥ 60 years using endovascular registry data from January 2018 to March 2022. We examined patient background, aneurysm characteristics, adjuvant technique, symptomatic complications, and mRS scores.

Results

The average age of the patients was 72.5±6.9 years, and 83 cases (74.1%) were female. During the postoperative follow-up period (six to 36 months), no deaths occurred, one case of postoperative rupture was observed, and nine patients (5.4%) were re-treated. Notably, age, underlying disease, aneurysm location, and re-treatment were not associated with complications. In the multivariate logistic analysis for re-treatment, symptomatic complications [odds ratio (OR) 11.01; 95% confidence interval (CI), 3.68-52.5; p < 0.001] and re-treatment (OR 3.25; 95% CI, 1.04-10.7; p = 0.039) were independently associated with mRS score deterioration. The risk factors for re-treatment were maximum aneurysm diameter and aneurysm neck diameter; aneurysms with neck diameters and maximum diameters > 5.0 mm and > 9.0 mm, respectively, had a higher rate of need for re-treatment (33%) and mRS score deterioration (33%) due to re-enlargement of the aneurysm.

Conclusion

In this study, complications did not increase with age in those aged 60 and older. However, prioritizing the avoidance of complications in elderly patients is important. Elderly patients with aneurysms larger than 9 mm have a poor prognosis and require additional attention for re-treatment.

## Introduction

The global population is aging, particularly in Japan, which has one of the longest life expectancies worldwide. The number of elderly patients with unruptured aneurysms is also increasing [1]. The risk of rupture is higher in elderly patients > 70 years and is further increased in those > 80 years [2]. As life expectancy increases, the number of elderly patients with unruptured cerebral aneurysms requiring treatment will also increase. The risks of perioperative cerebral infarction and functional complications are significantly higher in elderly patients than in young patients [3,4].

According to the American Heart Association/American Society of Anesthesiologists guidelines, observation is a reasonable alternative for patients aged > 65 years [5]. Regarding endovascular treatment of unruptured cerebral aneurysms in elderly patients, the risk factors for complications and prognosis are yet to be adequately reported. Therefore, we aimed to identify the outcomes of coiling in elderly patients and the risk factors for complications and prognosis.

## Materials and methods

Study design and patients population

This retrospective multicenter cohort study analyzed data from the Stroke Registry, including patients who underwent endovascular treatment for unruptured cerebral aneurysms at three facilities including a university hospital and a stroke center, between January 2018 and March 2022. The study aimed to identify prognostic factors and risk factors associated with complications during embolization of unruptured aneurysms in elderly patients. Elderly patients were defined as those > 60 years old [6]. As per the Personal Information Protection Law and National Research Ethics Guidelines in Japan, the participants were offered an opt-out option. This study was conducted according to the ethical standards of the Declaration of Helsinki and its subsequent amendments. We excluded certain patients based on the following criteria: lack of follow-up imaging with angiography or magnetic resonance angiography (MRA), a follow-up period of less than six months, treatment involving flow diverters (FD), the presence of mycotic or inflammatory aneurysms, and coexisting cerebral arteriovenous malformations or fistulas. After excluding those with insufficient data (18 cases), we analyzed data from 112 elderly patients.

The patient characteristics collected included age, sex, 90-day modified Rankin Scale (mRS) scores [7], comorbidities (diabetes, hypertension, chronic kidney disease, estimated glomerular filtration rate < 30 ml/min/1.73 m2, and hyperlipidemia), anti-platelet agents, aneurysm characteristics {aneurysm location and maximum diameter, neck diameter, dome-neck ratio, bleb, and scores for rupture risk factors, following the Unruptured Cerebral Aneurysm Study (UCAS) in Japan [8]}. Procedural factors and adjuvant techniques (balloon, stent-assisted, or double catheter) were also analyzed.

Angiographic results and follow-up evaluation

Four neurosurgeons classified the immediate postprocedural angiographic results as complete occlusion, neck remnant, and body filling. Magnetic resonance imaging (MRI) and magnetic resonance angiography (MRA) were conducted at one day, three to six months, and one year after coiling, followed by annual examinations. Cerebral angiography was performed when aneurysmal recanalization was suspected based on MRA findings. Re-treatment was performed when the space of the recurrent aneurysm was large enough for re-treatment or when it tended to increase.

Outcome measures

The outcome measures included mRS scores, which were evaluated preoperatively, 90 days after endovascular treatment, and at the time of any retreatment. Patient status at the final follow-up was determined through telephone interviews, outpatient visits, or data retrieved from electronic medical records. Complications associated with coiling were also analyzed. These complications were classified into symptomatic ischemia, identified by high signal intensity on diffusion-weighted MRI sequences, intracranial hemorrhage, neurological deficits, and systemic complications. Additionally, patients were grouped based on the change in mRS scores from preoperative to 90 days postoperative: those whose mRS scores decreased by one or more points were classified into the deterioration group.

Statistical analysis

We performed the statistical analysis after completing the study period to account for bias. The results are presented as the mean and standard deviation for quantitative data and as frequencies (percentages) for categorical data. The data were not normalized due to the limited number of enrolled patients. Pearson’s chi-square test (or Fisher’s exact test) and Wilcoxon tests were used to compare differences between groups. Significant variations in clinical variables, aneurysm characteristics, and endovascular treatment parameters among the groups were identified using a multivariate regression model. Odds ratios (ORs) and 95% confidence intervals (CI) were calculated. The model’s predictive ability was tested using the area under the receiver operating characteristic (ROC) curve. An area under the curve (AUC) of 0.70-0.79, 0.80-0.89, and 0.90-1.00 indicated average, good, and excellent discrimination, respectively. Statistical significance was set at p < 0.05. All statistical analyses were performed using the JMP 15 software (SAS Institute Inc., Cary, NC, USA).

## Results

Patient characteristics

This retrospective study evaluated 198 consecutive endovascular procedures for unruptured cerebral aneurysms. Of these, 130 aneurysms were identified in elderly patients. Eighteen cases were excluded from the analysis due to the absence of follow-up imaging or the use of an FD as treatment. Finally, 112 patients with sufficient data were included in this study. The mean age of the patients was 72.5 ± 6.9 years, with a median UCAS score of 6. Embolization was performed in 29 men (25.9%) and 83 women (74.1%). No deaths occurred, and the median preoperative and postoperative mRS scores were both zero. During the postoperative follow-up period (six to 36 months), one case of postoperative rupture was observed, and nine patients (8.0%) were re-treated. Table 1 shows the patient characteristics according to age. The proportion of women (60s, 70s, 80s and older:59.5%, 79.6%, and 93.8%; p = 0.013), symptomatic aneurysms (60s, 70s, 80s and older: 0%, 5.6%, and 18.8%; p = 0.018), internal carotid-posterior communicating arteries (IC-PC) (60s, 70s, 80s and older: 14.3%, 40.7%, and 81.3%; p < 0.001), and UCAS scores (60s, 70s, 80s and older: 4, 6, and 7.5 (median); p < 0.001) all tended to increase with increasing age.

**Table 1:** Endovascular treatment for unruptured aneurysm in elderly people Data are presented as the number (%), median (range),  or the mean (± standard deviation) CKD: chronic kidney disease, GFR: glomerular filtration rate, ICA: internal carotid artery, ICPC: internal carotid-posterior communicating artery, ACA: anterior cerebral artery, Acom: anterior communicating artery, MCA: medial cerebral artery, SD: standard deviation, UCAS: Unruptured Cerebral Aneurysm Study of Japan, DAC: distal access catheter, mRS: modified Ranking Scale.

characteristics	All Patients (n = 112)	60-69 years (N = 42)	70-79 years (N = 54)	over 80 years (N = 16)	p value
Women, n (%)	83 (74.1)	25 (59.5)	43 (79.6)	15(93.8)	0.013
Hypertension, n (%)	68 (60.7)	23 (54.8)	32 (59.3)	13 (81.3)	0.174
Diabetes melllitus, n (%)	7 (6.3)	4 (9.5)	3 (5.6)	0	0.390
Hyperlipidemia, n (%)	44 (39.3)	18 (42.9)	19 (35.2)	7 (43.8)	0.691
CKD (eGFR < 30 ml/min/1.73m2), n (%)	6 (5.4)	3 (7.1)	2 (3.7)	1 (6.3)	0.748
Aneurysm characteristic					
Symptomatic aneurysm, n (%)	6 (5.4)	0	3 (5.6)	3 (18.8)	0.018
Bleb, n (%)	39 (34.8)	16 (38.1)	18 (33.3)	5 (31.3)	0.843
Location of aneurysms, n (%)					
ICA	24 (21.4)	14 (33.3)	9 (16.7)	1 (6.3)	0.040
ICPC	41 (36.6)	6 (14.3)	22 (40.7)	13 (81.3)	< 0.001
ACA	2 (1.8)	1 (2.4)	1( 1.9)	0	0.828
Acom	13 (11.6)	7 (16.7)	6 (11.1)	0	0.206
MCA	9 (8.0)	4 (9.5)	5 (9.3)	0	0.442
Posterior circulation artery	21 (18.8)	10 (23.8)	10 (18.5)	1 (6.3)	0.309
Aneurysm size, mean (SD), mm	7.5 (±3.7)	6.7 (±3.3)	8.0 (±4.0)	8.0 (±4.0)	0.128
Mean neck, (SD), mm	4.1 (±2.6)	3.6 (±1.8)	4.6 (±3.1)	3.7 (±2.2)	0.332
Mean dome/neck, (SD)	1.7 (±0.9)	1.6 (±1.0)	1.7 (±0.6)	2.1 (±1.2)	0.298
Aneurysm morphology, n (%)					0.798
Saccular	106 (93.8)	39 (92.9)	51 (94.4)	15 (93.8)	-
Fusiform	3 (2.7)	1 (2.4)	1 (1.9)	1 (6.3)	-
Dissection	4 (3.6)	2 (4.8)	2 (3.7)	0 (0)	-
UCAS score, Median (range)	6 (1-12)	4 (1-11)	6 (2-11)	7.5 (3-12)	<0.001
Outcomes					
Treatment procedure, n (%)					
DAC	28 (25.0)	7 (16.7)	18 (33.3)	3 (18.8)	0.143
Balloon assist	35 (31.3)	10 (23.8)	18 (33.3)	7 (43.8)	0.308
Double catheter	7 (6.3)	1 (2.4)	3 (5.6)	3 (18.8)	0.068
Stent assist	36 (32.1)	15 (35.7)	17 (31.5)	4 (25.0)	0.730
Complete occlusion, n (%)	36 (32.1)	13 (31.0)	17 (31.5)	6 (37.5)	0.883
Complication, n (%)	24 (21.4)	7 (16.7)	13 (24.1)	4 (25.0)	0.634
Bleeding complication, n (%)	1 (0.9)	1 (2.4)	0	0	0.431
Ischemic complication, n (%)	16 (14.3)	5 (11.9)	9 (16.7)	2 (12.5)	0.784
Neurologic deficit, n (%)	3 (2.7)	1 (2.4)	1 (1.9)	1 (6.3)	0.625
Systemic complication, n (%)	0	0	0	0	-
Symptomatic complication, n (%)	9 (8.0)	4 (9.5)	4 (7.4)	1 (6.3)	0.894
PreEVT mRS					0.0044
0	76 (67.9)	35 (83.3)	36 (66.7)	5 (31.3)	-
1	20 (17.9)	5 (11.9)	10 (18.5)	5 (31.3)	-
2	11 (9.8)	0	6 (11.1)	5 (31.3)	-
3	2 (1.8)	1 (2.4)	0	1 (6.3)	-
4	3 (2.7)	1 (2.4)	2 (3.7)	0	-
PostEVT mRS 0-2, n (%)	107 (95.5)	40 (95.2)	52 (96.3)	15 (93.8)	0.904
Mean follow-up period, (±SD), month	10.1 (±4.2)	10.5 (±4.1)	9.8 (±3.8)	10.3 (±5.5)	0.779
Deterioration of mRS at last follow-up, n (%)	11 (9.8)	3 (7.1)	6 (11.1)	2 (12.5)	0.752
Retreatment after coiling, n (%)	9 (8.0)	6 (14.2)	2 (3.7)	1 (6.3)	0.748
Ruptured aneurysm after coiling, n (%)	1 (0.9)	1 (2.4)	0	0	0.431

Coiling and adjuvant techniques and outcome

Electrophysiological monitoring was not performed during coiling, and we used two or more coils for all the aneurysm embolization procedures. Nine of the 112 patients required re-treatment due to recurrence or coil compaction during follow-up. Among the nine re-treated cases, only one case (an 82-year-old woman with a maximum mass diameter of 9 mm) required re-treatment after achieving complete occlusion. We coiled 36 (32.1%, 36/112) wide-neck aneurysms (neck > 4 mm or dome-neck ratio < 2) using a stent. The other 76 cases consisted of aneurysms with a dome-to-neck ratio of less than 2, which were managed using various neck-remodeling techniques: 35 (31.3%, 35/112) balloon-remodeling cases and seven (6.3%, 7/112) multiple-catheter technique cases. We performed 34 operations (30.4%, 34/112) using a simple technique. Distal access catheters were used in 28 patients. None of the patients were treated with FDs. Table 1 presents the univariate analysis results of patient outcomes according to the age group. Among the elderly, no obvious differences per decade were observed, except for the percentage of preoperative mRS. In older age groups, the mRS0 proportion decreased while the mRS1 and mRS2 proportions increased (p = 0.0044).

Univariate and multivariate analyses for predictors of outcomes (deterioration of mRS)

Table 2 presents the univariate and multivariate analyses results of mRS score deterioration after coiling. mRS scores deteriorated after coil embolization in 11 patients. mRS scores worsened in approximately 50% of the patients due to complications (6/11 cases, 54.6%), but < 50% had worsening mRS scores due to neurological symptoms associated with enlarged aneurysms (5/11 cases, 45.4%). The mRS score decreased by over two points in one patient. The analysis of mRS score deterioration showed that symptomatic complication (OR, 11.0; 95% CI, 3.68-52.5; p < 0.001) and re-treatment after coiling (OR, 3.25; 95% CI, 1.04-10.7; p = 0.039) were independently associated with mRS score deterioration.

**Table 2:** Univariate analysis for deterioration of mRS Data are presented as the number (%)  or the mean (± standard deviation) *: Fisher exact test, †: unit odds ratio, CKD: chronic kidney disease, GFR: glomerular filtration rate, ICA: internal carotid artery, ICPC: internal carotid-posterior communicating artery, ACA: anterior cerebral artery, Acom: anterior communicating artery, MCA: medial cerebral artery, SD: standard deviation, UCAS: Unruptured Cerebral Aneurysm Study of Japan, DAC: distal access catheter, mRS: modified Ranking Scale.

variate	deterioration of mRS		univariate	multivariate	
	Yes, n = 11	No, n = 101	p value	Odd (95% CI)	p value
Age mean±SD years	74.2 ± 6.3	72.2 ± 7.3	0.323	1.16† (1.01-1.37)	0.051
Women, n (%)	9 (81.8)	74 (73.3)	0.725*	0.78† (0.20-3.35)	0.712
Hypertension, n (%)	7 (63.6)	60 (59.4)	0.999*	-	-
Diabetes melllitus, n (%)	0	7 (6.9)	0.999*	-	-
Hyperlipidemia, n (%)	6 (54.6)	38 (37.6)	0.275	-	-
CKD (eGFR < 30 ml/min/1.73m2), n (%)	0	5 (5.0)	0.999*	-	-
Haemodialysis, n (%)	0	4 (4.0)	0.999*	-	-
Heart disease, n (%)	1 (9.1)	6 (5.9)	0.525*	-	-
Aorta aneurysm, n (%)	0	3 (3.0)	0.999*	-	-
Tortuosity of access route, n (%)	0	11 (9.8)	0.597*	-	-
Aneurysm characteristic n (%)					
Symptomatic aneurysm	2 (18.2)	4 (4.0)	0.106*	-	-
Bleb	2 (18.2)	34 (33.7)	0.498*	-	-
Location of aneurysms, n (%)					
ICA	4 (36.7)	20 (19.8)	0.245*	-	-
ICPC	4 (36.4)	37 (36.6)	0.999*	-	-
ACA	0	2 (2.0)	0.999*	-	-
Acom	0	13 (12.9)	0.357*	-	-
MCA	0	9 (8.9)	0.595*	-	-
Posterior circulation artery	3 (27.2)	18 (17.8)	0.429*	-	-
Maximum diameter Mean ±SD (mm)	10.7 ± 5.2	7.3 ± 3.5	0.035	1.15† (0.88-1.52)	0.303
Neck diameter Mean ±SD (mm)	6.2 ± 4.4	3.9 ± 2.1	0.076	-	-
Dome/neck mean±SD	1.41 ± 0.79	1.78 ± 0.89	0.084	-	-
UCAS score, Median (range)	6 (5-9)	5 (1-12)	0.046	0.98† (0.56-1.66)	0.934
Treatment procedure, n (%)					
DAC	2 (22.2)	26 (25.2)	0.999*	-	-
Balloon assist	2 (18.2)	32 (31.7)	0.499*	-	-
Double catheter	1 (9.1)	6 (5.9)	0.682*	-	-
Stent assist	5 (45.5)	33 (32.7)	0.395	-	-
Complete occlusion, n (%)	4 (36.4)	33 (32.7)	0.999*	-	-
Symptomatic complication, n (%)	6 (54.6)	3 (3.0)	< 0.001*	11.01 (3.68-52.51)	< 0.001
Retreatment after coiling, n(%)	3 (27.3)	6 (5.9)	0.043*	3.25 (1.04-10.72)	0.039

The analyses for predictors of re-treatment after coiling

**Figure 1:**
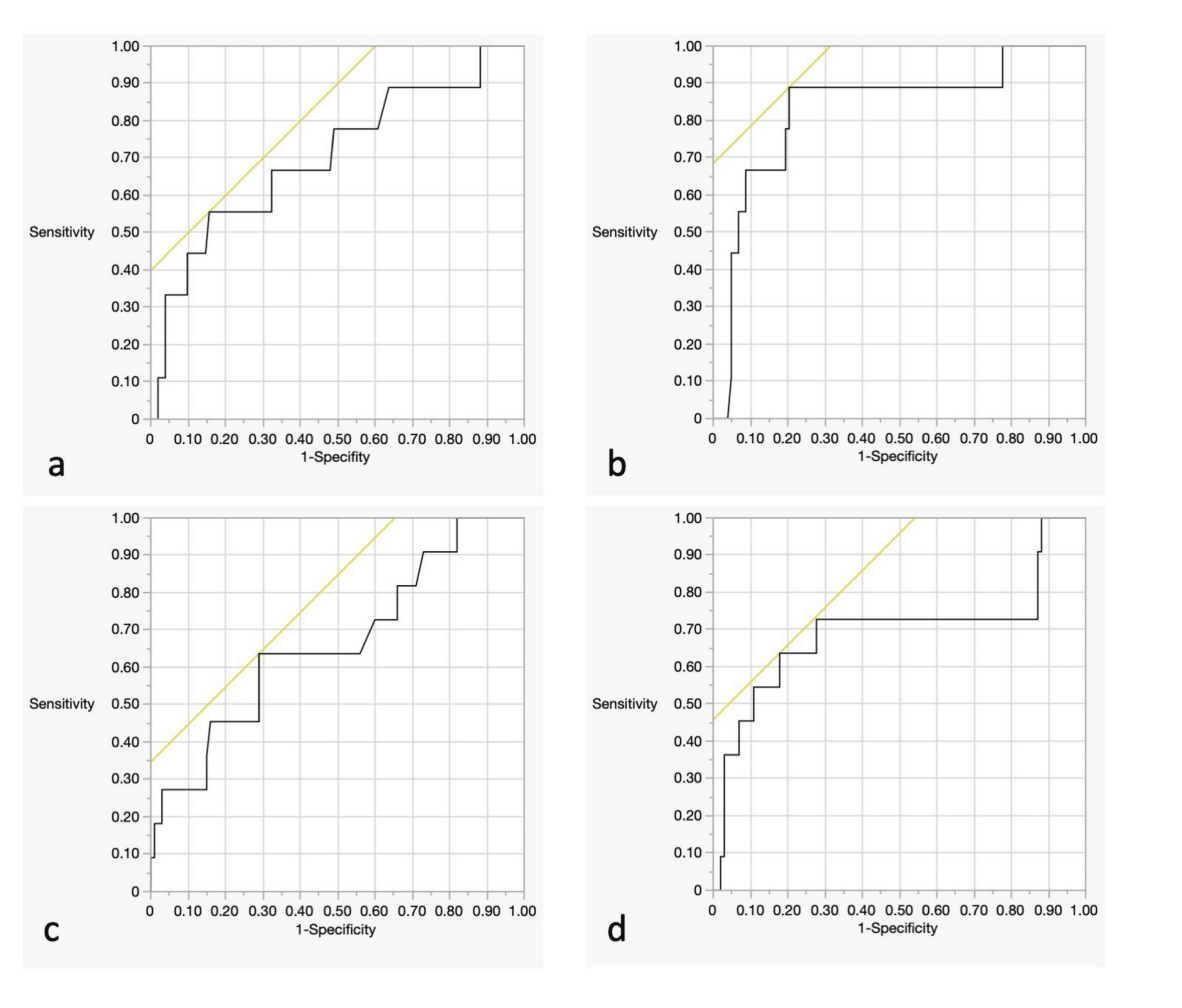
The receiver operating characteristic(ROC) curve analysis of re-treatment and deterioration of mRS a: ROC analysis of neck diameter for re-treatment was shown. The ideal cut-off value of neck diameter was 5.6 mm, and the area under the curve (AUC), sensitivity, and specificity were 0.704, 0556., and 0.876, respectively. b: ROC analysis of maximum aneurysm diameter for re-treatment was shown. The ideal cut-off value of the aneurysm diameter was 9.2 mm, and the AUC, sensitivity, and specificity were 0.831, 0.889, and 0.796, respectively. c: ROC analysis of neck diameter for deterioration of mRS was shown. The ideal cut-off value of neck diameter was 4.4 mm, and the AUC, sensitivity, and specificity were 0.663, 0.710, and 0.636, respectively. d: ROC analysis of maximum aneurysm diameter for deterioration of mRS was shown. The ideal cut-off value of the aneurysm diameter was 9.7mm, and the AUC, sensitivity, and specificity were 0.693, 0.822, and 0.636, respectively.

**Figure 2:**
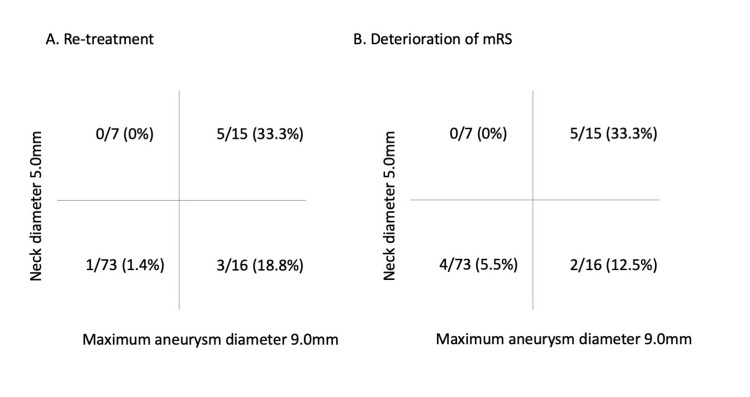
Prediction diagram of re-treatment and Deterioration of mRS by maximum aneurysm diameter > 9.0mm and neck diameter > 5.0mm (A) An age > 60 years, maximum aneurysm diameter > 9.0 mm and neck diameter > 5.0mm yielded a re-treatment rate of 33%, (B) An age > 60 years, maximum aneurysm diameter > 9.0 mm and neck diameter > 5.0 mm yielded an mRS deterioration rate of 33%.

During follow-up, recurrence was observed in nine patients (8.0%) requiring re-treatment. Table 3 presents the univariate analysis results of re-treatment after coiling. There were 37 cases of complete occlusion (33.0%), and of these 37 cases, only two were re-treated for recurrence. The analysis of re-treatment after coiling showed that maximum aneurysm diameter (re-treated vs. not re-treated cases; 11.9±3.6 vs. 7.3±3.6 mm; p = 0.001) and aneurysm neck diameter (re-treated vs. not re-treated cases; 5.9±3.2 vs. 4.0±2.4 mm; p = 0.043) were associated with re-treatment. The cutoff values associated with re-treatment of aneurysm neck diameter and maximum aneurysm diameter were determined using the ROC analysis. The aneurysm neck diameter showed good discrimination, with an AUC of 0.876. The cutoff value for the aneurysm neck diameter was 5.6 mm, and the sensitivity and specificity were 0.704 and 0.556, respectively (Figure 1a). The maximum aneurysm diameter showed average discrimination, with an AUC of 0.796. The cutoff value for the maximum aneurysm diameter was 9.2 mm, and the sensitivity and specificity were 0.831 and 0.889, respectively (Figure 1b). Similarly, the cutoff values for aneurysm size and neck size were 4.4 mm (AUC: 0.636) and 9.7 mm (AUC: 0.636), respectively, when examined with respect to mRS score deterioration (Figures 1c, 1d). Based on these cutoff values, when the case group is divided into four groups according to an aneurysm neck diameter of 5.0 mm and a maximum aneurysm diameter of 9 mm, elderly patients with neck diameters > 5.0 mm and maximum aneurysm diameters > 9 mm exhibit a higher rate of re-treatment (33%) and mRS score deterioration (33%) (Figure 2).

**Table 3:** Univariate analysis of retreatment Data are presented as the number (%)  or the mean (± standard deviation) *: Fisher exact test, CKD: chronic kidney disease, GFR: glomerular filtration rate, ICA: internal carotid artery, ICPC: internal carotid-posterior communicating artery, ACA: anterior cerebral artery, Acom: anterior communicating artery, MCA: medial cerebral artery, SD: standard deviation, UCAS: Unruptured Cerebral Aneurysm Study of Japan, DAC: distal access catheter, mRS: modified Ranking Scale,

variate	retreatment		univariate
	Yes, n = 9	No, n = 103	p value
Age mean±SD years	70.2 ± 6.6	72.6 ± 7.3	0.324
Women, n (%)	5 (55.6)	78 (75.7)	0.234
Hypertension, n (%)	5 (55.6)	62 (60.2)	0.999*
Diabetes melllitus, n (%)	1 (11.1)	6 (5.8)	0.453
Hyperlipidemia, n (%)	4 (44.4)	40 (38.8)	0.736
CKD (eGFR < 30 ml/min/1.73m2), n (%)	0	5 (4.9)	0.999*
Haemodialysis, n (%)	0	4 (3.9)	0.999*
Heart disease, n (%)	1 (11.1)	6 (5.8)	0.453*
Aorta aneurysm, n (%)	0	3 (2.9)	0.999*
Tortuosity of access route, n (%)	0	11 (10.7)	0.595*
Aneurysm characteristic, n (%)			
Symptomatic aneurysm	1 (11.1)	5 (4.9)	0.402*
Bleb	3 (33.3)	33 (35.0)	0.999*
Location of aneurysms, n (%)			
ICA	2 (22.2)	22 (21.4)	0.999*
ICPC	3 (33.3)	38 (36.9)	0.999*
ACA	0	2 (1.9)	0.999*
Acom	0	13 (12.6)	0.595*
MCA	0	9 (8.7)	0.999*
Posterior circulation artery	4 (44.4)	17 (16.5)	0.062*
Maximum diameter Mean ±SD (mm)	11.9 ± 3.6	7.3 ± 3.6	0.001
Neck diameter Mean ±SD (mm)	5.9 ± 3.2	4.0 ± 2.4	0.043
Dome/neck Mean±SD	2.1 ± 0.72	1.72 ± 0.90	0.157
Treatment procedure, n (%)			
DAC	2 (22.2)	27 (26.2)	0.999*
Balloon assist	3 (33.3)	31 (30.1)	0.999*
Double catheter	0	7 (6.8)	0.999*
Stent assist	4 (44.4)	34 (33.0)	0.485
Complete occlusion, n (%)	2 (22.2)	35 (34.0)	0.715*
Symptomatic complication, n (%)	1 (11.1)	8 (7.8)	0.543

## Discussion

This retrospective, three-center, observational study examined consecutive elderly patients who underwent coiling for unruptured aneurysms. No significant differences were observed in complications according to the age group. Careful indications and treatments should be provided to older patients. Elderly patients with wide necks > 5.0 mm and maximum aneurysm diameters > 9.0 mm are associated with complication-independent mRS score deterioration and re-treatment. To the best of our knowledge, no previous reports have reported age as a factor related to re-treatment in unruptured aneurysms, nor have any reports examined unruptured aneurysm size and prognosis (change in mRS, not risk of rupture). This is the first report to suggest that aneurysm size may affect mRS in elderly patients due to aneurysm growth, independent of rupture. The ability to predict the risk of re-treatment after coiling is useful in planning treatment strategies.

The risks and benefits should be considered when treating unruptured aneurysms in elderly patients. A previous study showed that the risk of perioperative cerebral infarction was significantly higher in elderly patients than in young patients [3]. However, the global life expectancy and the risk of rupture are increasing. Hishikawa et al. reported an annual rupture rate of 1.6% for patients aged ≥ 70 years, with a particularly high rupture rate of > 3% for those aged ≥ 80 years [2]. The high rupture rate of large aneurysms was also evident in the International Study of Unruptured Intracranial Aneurysms (ISUIA) and may justify treating unruptured aneurysms in elderly patients [9]. Therefore, the benefit of treatment for unruptured aneurysms in elderly patients at a high risk of rupture is relatively high. Even in such cases, a comparison of the lifetime rupture rate with the surgical complication rate is an important criterion for treatment [10]. The indications for treating unruptured aneurysms in elderly patients should be determined on an individual basis, taking into consideration several factors, including an accurate understanding of the natural history, risks associated with treatment, comorbidities, life expectancy, and patient and family preferences.

This study found that elderly patients aged > 80 years had symptomatic aneurysms. In the UCAS Japan study, large aneurysms were found significantly more frequently in elderly patients, with a 39.7% rate among those > 80 years of age [1]. In addition, the incidence of posterior communicating artery (PcomA) aneurysms continues to increase in elderly patients [11]. Therefore, we found a higher percentage of IC-PC aneurysms in elderly patients in this study. In addition, older patients with a higher preoperative mRS score were treated. Reportedly, preoperative clinical conditions strongly correlate with postoperative clinical outcomes [12]. The treatment outcome may be related to the quality of the preoperative clinical condition rather than advanced age.

Mahaney et al. [13] reported from a sub-analysis of 4059 patients in the ISUIA study that clipping was associated with a higher incidence of surgical and postoperative complications, such as cerebral hemorrhage, stroke, sepsis, and epilepsy in patients aged ≥ 65 years (19.0%) compared to the endovascular group (8.0%). A study of the National Inpatient Sample in the United States also showed that patients aged 65-79 years who underwent coil embolization had significantly lower morbidity (6.9% vs. 26.8%) and mortality (0.8% vs. 2.0%) rates than those who underwent clipping [14]. Hishikawa et al. suggested that elderly patients with aneurysms > 7 mm or those with IC-PC aneurysms may be suitable candidates for coil embolization if it can be safely performed [10]. Therefore, we considered endovascular treatment as the first choice for elderly patients.

There have been several reports on the results of endovascular treatment for elderly patients with unruptured cerebral aneurysms, and many reports have shown no difference in the safety of treatment compared with non-elderly patients [15], with good clinical results [16]. Perioperative complications were more frequent than those in previous meta-analyses, especially in patients aged > 80 years [17]. In our study, we found no significant differences in complications among patients aged ≥ 60 years, including those in their 80s. The ISUIA study reported an overall morbidity and mortality rate of 9.3% for patients undergoing endovascular coiling [9]. This rate is comparable to our current complication rate of 9.8%. Additionally, previous research, which was not limited to elderly populations, has documented complication rates ranging from 5% to 10%[18-20]. Furthermore, the ISUIA study did not identify age as a significant predictor of poor outcomes in its endovascular cohort [9]. This may be due to the policy of placing safety first as a treatment strategy for the elderly.

In this study, unfavorable outcomes after treatment correlated significantly with re-treatment. It has been suggested that preventing symptomatic complications during the initial treatment leads to re-treatment prevention. In addition, cases of incomplete coil occlusion of large aneurysms are prone to re-enlargement and re-rupture [21,22]. However, as shown in our study, symptomatic complications have the strongest prognostic impact. Complicated procedures such as forcing complete occlusion or using a stent may trigger complications [23]. Therefore, in elderly patients prone to surgical complications, evacuation treatment to avoid complications and reduce the risk of rupture is considered an option rather than aiming for a radical cure of the aneurysm [21], even if there is a risk of recurrence. FD therapy is currently used to treat difficult-to-treat unruptured aneurysms. FD has been reported to safely and significantly reduce operative time for treating wide-neck aneurysms [24]. Brinjikji et al. reported that the overall complication rate for flow diverter (FD) therapy in carefully selected patients aged > 70 years was relatively low, suggesting that age alone should not be a contraindication for FD therapy [25]. FDs may have lower complete occlusion (CO) rates in the elderly [26]. Notably, two patients who were > 70 years old were treated for FD in our registry but had transient ischemic complications, and CO was not obtained at one-year follow-up; however, they were excluded from this study. In our study, the aneurysms treated in elderly patients were large IC-PC aneurysms, and most cases had a PcomA incorporated into the aneurysm. Previous studies indicated that aneurysms with incorporated branches are less likely to result in complete occlusion [27]. In particular, fetal PComA aneurysms are refractory to FD treatment [28,29]. Therefore, large symptomatic IC-Pcom aneurysms present significant challenges in elderly patients. However, issues regarding the elderly remain unclear, and further studies are needed. Elderly patients often have various background factors; hence, they may be at a high risk of bleeding due to double antiplatelet agents, and it is necessary to find the most suitable individualized treatment.

Limitations

This study has a few limitations. All patients were Japanese adults selected from a registry database comprising three hospitals. Cases were identified retrospectively in multiple centers, which resulted in a clear selection bias for unruptured cases because treatment was proposed mainly if the aneurysm morphology seemed suitable for low-risk endovascular interventions. No comparisons were made with younger patients or with patients who did not undergo any interventions. We could not examine whether the treatment for elderly patients was as safe as that for younger patients. The observation period was short; however, it has been useful for confirming treatment outcomes in recent years. In addition, some patients were excluded because their follow-up period was too short. Prolonging the observation period may further increase the number of cases with re-treatment or rupture. Therefore, further research on the long-term effects of unruptured cerebral aneurysms in elderly patients is needed.

## Conclusions

In this study, there was no increase in the number of complications associated with advanced age. Endovascular treatment was safely performed in elderly patients. Aneurysms with a higher risk of rupture are more likely to recur after coiling; thus, treatment strategies should be carefully considered, and avoiding complications should be a top priority in the elderly. In elderly patients with maximum aneurysm diameters > 9 mm, careful attention should be paid to the potential for neurological symptoms related to aneurysm regrowth after endovascular treatment to develop or worsen and the possibility that re-treatment might be required.
